# Screening for compounds released from deep brain stimulation probes by contact with a brain simulant

**DOI:** 10.1038/s41598-026-46292-5

**Published:** 2026-04-09

**Authors:** Yassine Bouattour, Jeremy Pinguet, David Bourgogne, Pierre-Olivier Bussière, Virgile Koubi, Bénédicte Mailhot-Jensen, Yoann Le Basle, Christelle Blavignac, Jean-Jacques Lemaire, Valérie Sautou

**Affiliations:** 1 CHU Clermont Ferrand, Clermont Auvergne INP, CNRS, ICCF, Université Clermont Auvergne, 63000 Clermont-Ferrand, France; 2 CHU Clermont-Ferrand, Pôle Pharmacie, 63003 Clermont-Ferrand, France; 3 Clermont Auvergne INP, CNRS, ICCF, Université Clermont Auvergne, 63000 Clermont–Ferrand, France; 4 Centre Imagerie Cellulaire Santé, UCA PARTNER, 63000 Clermont-Ferrand, France; 5 CNRS, Clermont Auvergne INP, Institut Pascal, Universite Clermont Auvergne, 63000 Clermont-Ferrand, France; 6 CHU Clermont Ferrand, Service de Neurochirurgie, 63000 Clermont-Ferrand, France

**Keywords:** Deep brain stimulation, Leachables, Extractables, Brain phantom, Medical devices, Chemistry, Materials science

## Abstract

Deep brain stimulation involves several neurological conditions in which implantable leads deliver electrical impulses to specific brain regions. While biocompatibility testing is essential for regulatory approval, leachables have not yet been identified. This study investigated extractables and leachables from polyurethane (PU)-insulated DBS probes, shedding light on their safety in clinical contexts. Two types of PUs, polyether (PEU) and polycarbonate urethane (PCU), were placed in contact with a brain phantom for 180 days at 37 °C. We performed an extraction using acetone and n-hexane on parts that were or were not in contact with the phantom to establish extractable profiles and to estimate leachable migration in the phantom. Analyses were performed via gas and liquid chromatography coupled with mass spectrometry and via inductively coupled plasma. Surface characterization was performed before and after contact to explain the differences in leachable migration between both PEU and PCU. The results revealed a decrease in the 4,4′-diphenylmethane diisocyanate concentration of 81.6% in the PEU and 62.3% in the PCU. Aluminum was quantifiable only for PEU (acetone extract) and decreased by 43.1% after contact with the simulant. This finding urges the need to conduct an evaluation of leachable migration in patients.

**Supplementary Information:**

The online version contains supplementary material available at 10.1038/s41598-026-46292-5.

## Introduction

Severe Parkinson’s disease, essential tremor, and dystonia are known neurological conditions improved by deep brain stimulation (DBS), a game-changing neurosurgical procedure. Flexible leads less than two millimeters in diameter are implanted into deep brain structures, such as the subthlamic nucleus, where they administer regulated high-frequency electrical impulses that modulate neuronal activity^1^. Clinical effectiveness has been demonstrated to enhance patients’ quality of life^2–4^. However, several side effects can occur, such as dysarthria, gait disorders and behavioral disorders^5^. The lead, often referred to as the “electrode”, commonly contains 4 to 8 distal electric contacts. The lead undergoes an assessment of biocompatibility before receiving marketing approval to meet safety requirements. The probes are made of platinum and iridium electrodes insulated by polycarbonate urethane (PCU) or polyether urethane (PEU) and remain in contact with the cerebral environment throughout the patient’s life^6^. Inflammatory chronic reactive gliosis, or scarring, develops around the probe^7–10^.

The study of leachables from DBS probes should be of upmost importance in evaluating their safety, particularly the biocompatibility of these medical devices. Manufacturers are required to carry out exhaustive tests to identify potential leachable substances and assess their risks for patient health in accordance with current regulations, such as the Food and Drug Administration (FDA) guidelines in the United States of America and the European Regulation 2017/745 in Europe^11,12^. However, the regulatory requirements are general and aim to ensure the safety of medical devices. Consequently, the tests carried out by manufacturers to obtain marketing authorization follow the biocompatibility standard ISO 10,993, which focuses on basic criteria, but they do not necessarily cover all the specificities linked to the use of DBS probes in a particular clinical context^13^. Indeed, parts 12 and 18 provide the framework allowing the study of extractable substances, i.e., the substances released by a medical device or a constituent material when the medical device or the material is brought into contact with solvents allowing forced extraction. However, unlike extractable substances, for which research methodologies are well defined, methods for studying leachable substances are still lacking. The search for leachable substances is considered only via a risk analysis based on data from the search for extractables, namely, a leachable search is carried out when the quantity of any extractable product released by the medical device presents a potential safety hazard owing to its estimated clinical release. The risk assessment linked to exposure is therefore based on the most unfavorable chemical release hypothesis with total exposure to the chemical constituents of the medical device and does not consider the migration kinetics and the quantities released^14^. As a result, this risk analysis approach does not consider substances whose toxicity does not have a dose‒effect relationship. Furthermore, in practice, the study of leachables can reveal the presence of substances that were not observed during a study of extractables^15–17^. Further study of substances released from these probes may provide insight into the specific interactions between the materials used in these probes and brain tissue. Therefore, it may be necessary to consider the unique characteristics of these devices, including the proximity of the probes to the brain and the particular physiological conditions to which they are subjected.

The identification of leachables in clinical settings, even after the granting of marketing authorization, can lead to increased regulatory surveillance or, in some cases, restrictions on use or withdrawal from the market. For example, cobalt release from certain orthopedic implants has led to reinforced labeling obligations (as cobalt has been classified as a CMR category 1B substance since 2019^18^) and enhanced postmarket monitoring by the French National Agency for the Safety of Medicines and Health Products. In another case, the Essure® intrauterine device was withdrawn from the French market, not because of cobalt but because of safety concerns, including the presence of tin^19^, highlighting the importance of ongoing vigilance. This shows the difficulties of the actual biocompatibility standard in avoiding all risks because of the lack of standardized and sometimes nonadapted methods for assessing leachables^20^. For DBS leads, polyurethane is composed of isocyanates, some of which are suspected to be carcinogenic, such as 4,4′-diphenylmethane diisocyanate (4,4′-MDI^21^. However, only one study has been conducted on extractable profiling in PCU probes, identifying the presence of only butylated hydroxytoluene but not other information about other components^22^. In addition, no studies have yet evaluated leachables from these intracerebral probes under realistic conditions. In this work, we aimed to identify extractables from two types of DBS probes, PEU and PCU, and to evaluate leachables by comparing extractables of probes without and after contact with a brain simulant for leachable screening.

## Results

### Extractable and leachable profiling

#### Gas chromatography–mass spectrometry

Chemicals found in scan mode are presented in supplementary data 2. The analysis of hexane and acetone extracts with gas chromatography–mass spectrometry (GC‒MS) revealed the presence of 4,4′-MDI and its isomers in both the PEU and PCU extracts, which is a suspected carcinogenic substance (class 2) according to the European CLP regulation. No other CMR or neurotoxic substances were found.

**Fig. 1:**
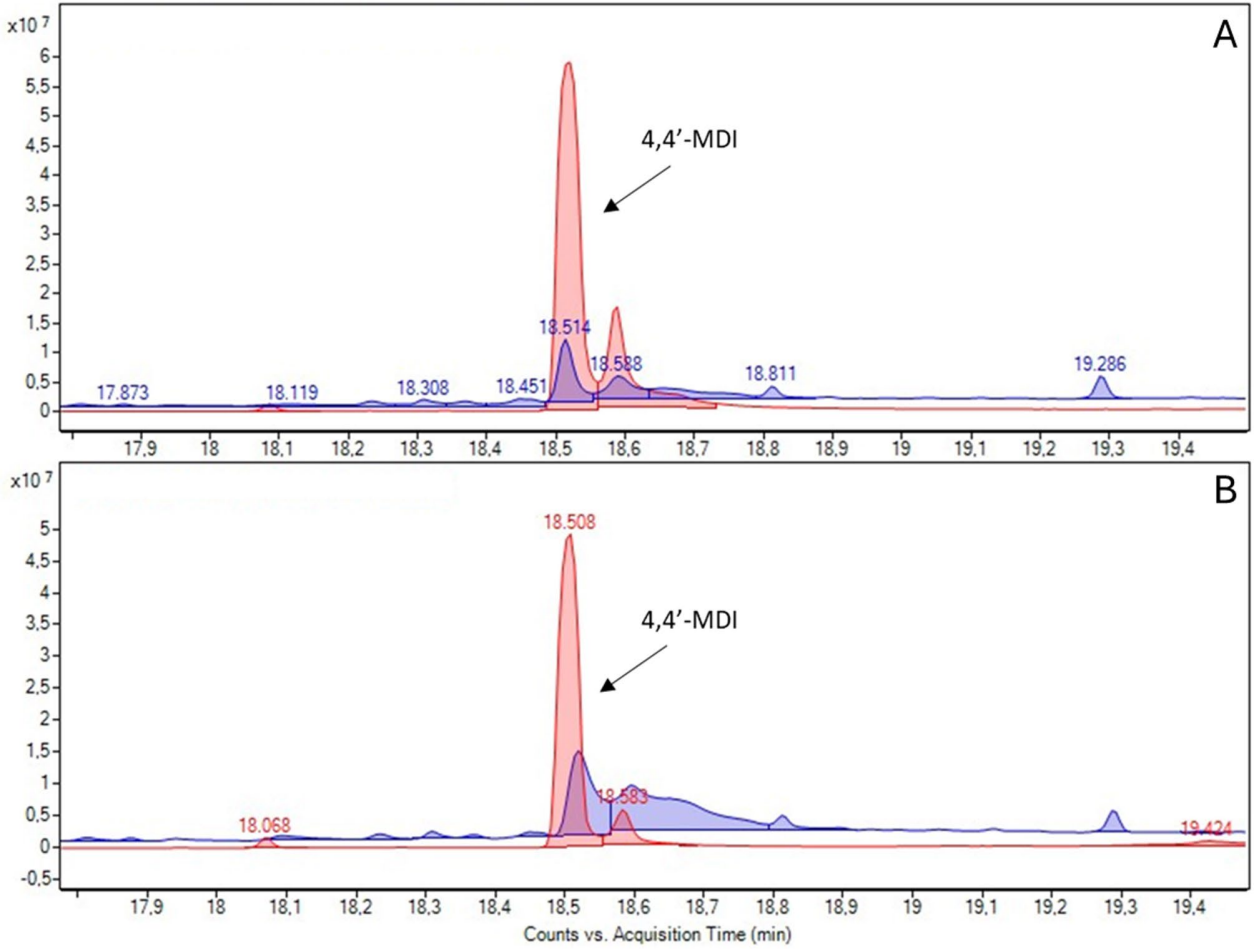
Variation of 4,4′-Methylene diphenyl diisocyanate (4,4′-MDI) analyzed by gas chromatography–mass spectrometry (scan mode) in acetone extract between no-simulant (red) and in-simulant (blue) polyether urethane (**A**) and polycarbonate urethane (**B**) leads.

The quantification of the 4,4′-MDI revealed a decrease in this chemical between the no-simulant and in-simulant leads, ranging from 1.52 mg per 100 mg of polyurethane (% w/w) to 0.26% w/w (82.6% decrease) and from 2.05 to 0.77% w/w (62.3% decrease) for the PEU and PCU, respectively (Fig. 1).

### Liquid chromatography–mass spectrometry

**Fig. 2:**
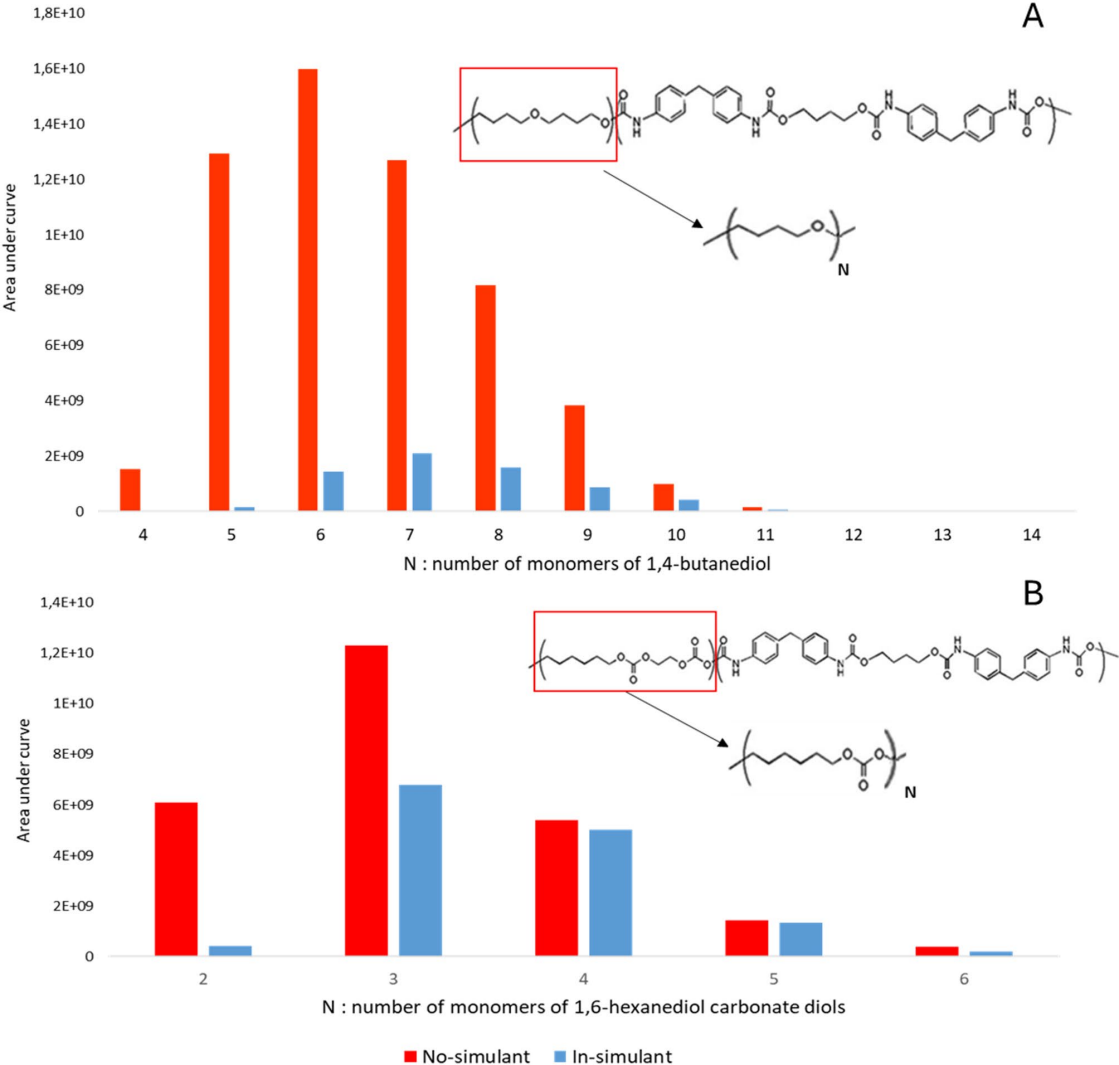
Area under the curve variation in response to oligomers of 1,4-butanediol in acetone extract between no-simulant (without contact with the simulant) and in-simulant (after contact with the simulant) polyether urethane lead.

The results of the liquid chromatography‒mass spectrometry (LC‒MS) analysis are presented in supplementary data 2. We found that PEU leads contain 1,4-butanediol oligomers in their acetone extracts, and PCU leads contain 1,6-hexanediol carbonate diol oligomers. For both polymers, we noticed a decrease in the area under the curves between the no-simulant and in-simulant leads in all oligomers, but especially in the low-n oligomers, reaching 99.04% for PEU and 92.98% for PCU (Fig. 2).

#### Inductively coupled plasma optical emission spectroscopy inductively coupled plasma optical emission spectroscopy

Among the 34 elements investigated via inductively coupled plasma optical emission spectroscopy (ICP‒OES) analysis, only Al and Cu were observed after the extraction of PEU and PCU. Al was not detectable in hexane, and its quantification was only possible in acetone. We found a decrease of 43.1% in the in-simulant PEU compared with the no-simulant polymer. The Al quantities, however, were not quantifiable for either in-simulant or no-simulant PCU lead since the concentration was below the limit of quantification. On the other hand, Cu was observed in both hexane and acetone, but the quantities were also not determined since its concentration was below the limit of quantification for both hexane and acetone. The results are presented in Table 1.

**Table 1:** Variations in the concentrations of elements obtained via inductively coupled plasma optical emission spectroscopy in extraction solvents for polyether (PEU) and polycarbonate (PCU) urethane; a: quantities with respect to the limit of detection; b: quantities with respect to the limit of quantification.

Element (extraction solvent)	PEU		PCU	
	No-simulant	In-simulant	No-simulant	In-simulant
Aluminum (acetone extraction) (ng/mg)	0.267	0.131	between 0.067^a^ and 0.133^b^	between 0.078^a^ and 0.135^b^
Copper (hexane extraction) (ng/mg)	between 0.061^a^ and 0.65^b^	between 0.071^a^ and 0.764^b^	< 0.05^a^	between 0.059^a^ and 0.545^b^
Copper (acetone extraction) (ng/mg)	between 0,048^a^ and 0.509^b^	between 0.05^a^ and 0.533^b^	between 0.05^a^ and 0.538^b^	between 0.059^a^ and 0.545^b^

### Spectral analysis and surface characterization

The IR spectrum of the PEU lead presented the characteristic 1111 cm^−1^ ether function band and the 3333 cm^−1^, 1533 cm^−1^ and 1225 cm^−1^ bands, which are characteristic of urethane function. We also found that the ratio of the 1730 cm^−1^ signal (free carboxyl function) to the 1700 cm^−1^ band (hydrogen-linked carboxyl function) was approximately 40 to 43%, confirming that the polymer was polyether urethane 80A^23,24^. On the other hand, the spectrum of the PCU probes presents a band at 3333 cm^−1^, 1533 cm^−1^ and 1225 cm^−1^, characteristic of urethane function; the 1736 cm^−1^ band corresponds to the carboxyl function of the carbonate groups; and the band at 1111 cm^−1^, characteristic of the ether function^23,25^. The comparison of the IR spectrum after 180 days of contact with the brain phantom did not reveal any changes for either the PEU or PCU probes (Supplementary material 1).

**Fig. 3:**
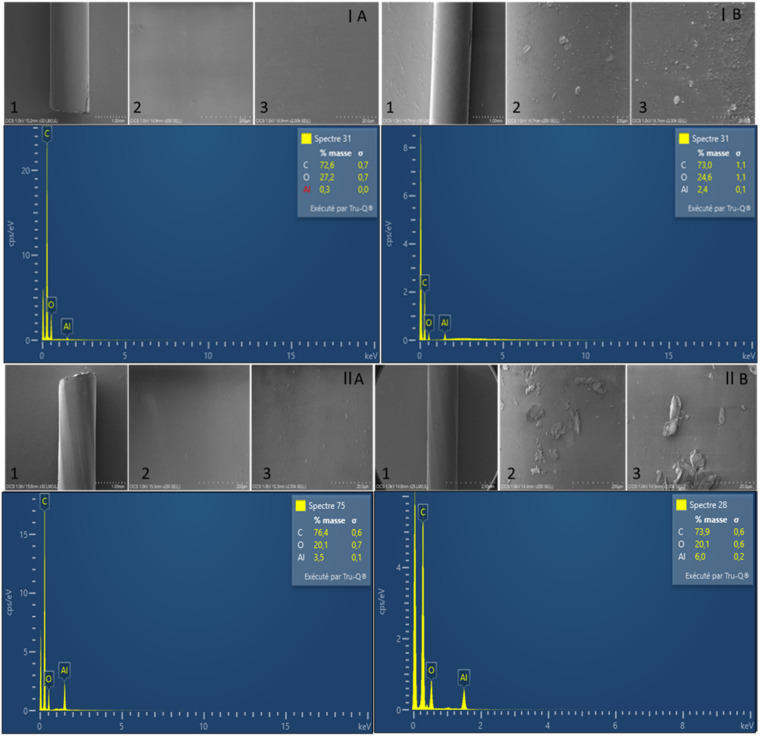
Scanning electron microscopy observations with representative energy-dispersive X-ray spectra for (**A**) no-simulant and (**B**) in-simulant polyether urethane (I) and polycarbonate urethane (II) leads with scale bars of 1000 (1), 200 (2) and 20 µm (3).

**Fig. 4:**
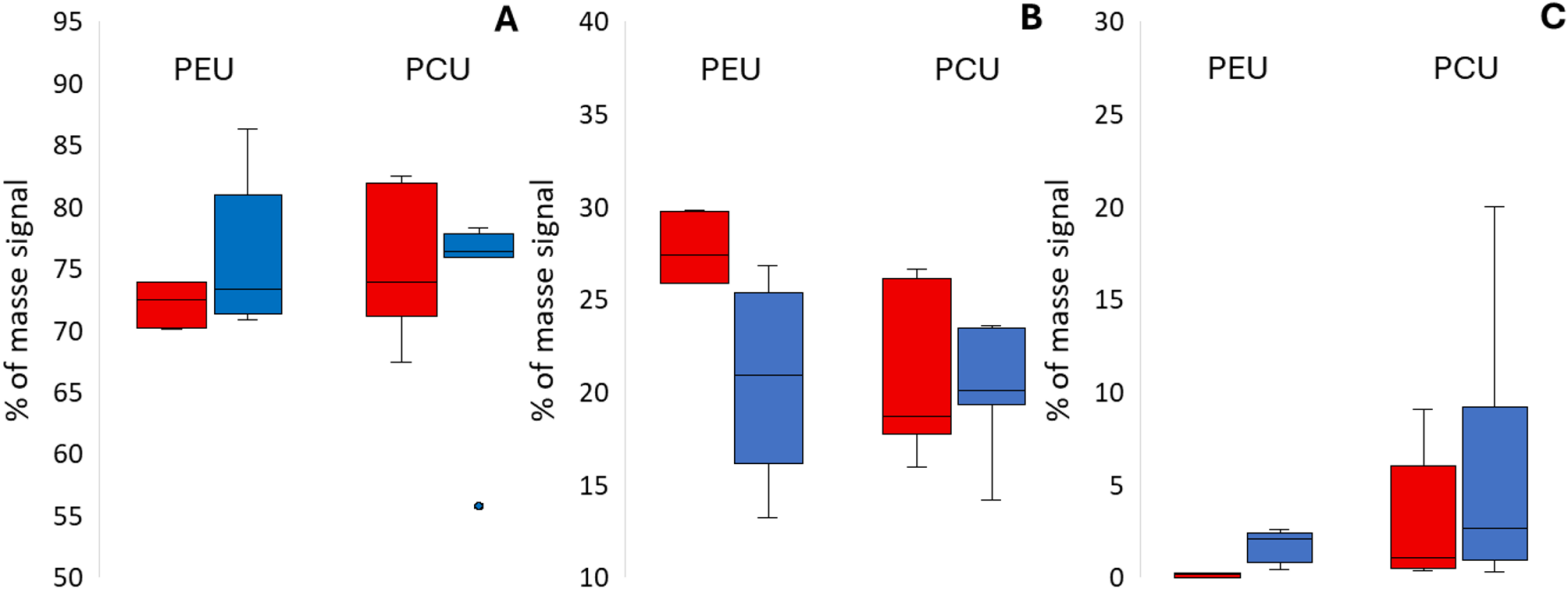
Elemental signal variation of (**A**) carbon, (**B**) oxygen and (**C**) aluminum between no-simulant (red) and in-simulant (blue) polyether urethane (PEU) and polycarbonate urethane (PCU) (n ≥ 6 surface locations per condition).

The scanning electron microscopy (SEM) observations revealed no cracking in either the PEU or PCU leads. However, we visually noticed increasing roughness on the surface of both polymers, which was more visible with PEU than with PCU (Fig. 3). Energy-dispersive X-ray spectroscopy (EDS) analysis revealed that the signal of Al was 10 times lower in the PEU polymer than in the PCU polymer. However, Al increased in the PEU lead, increasing from an average of 0.13% to 1.71% between the no-simulant and in-simulant samples, respectively. The same observation was noted for the PCU polymer containing a 30-fold greater signal of Al, with averages of 3.18% to 6.33% between the no-simulant and in-simulant samples, respectively (Fig. 4).

AFM analysis revealed variation in the surface texture between polymers without and after contact with the phantom for both PEU and PCU. We noticed the occurrence of valleys reaching a width of 25 nm (see Supplementary material 2). The roughness also increased in the PEU after contact with the brain phantom, but the variation was less apparent for the PCU polymer. Nanoindentation experiments revealed that for both PEU and PCU, the slope of both the loading and unloading curves decreased, implying a loss of rigidity of the polymer after 180 days of contact (Table 2).

**Table 2:** Variations in the quadratic roughness (Rq), approach and retraction slope between no-simulant and in-simulant polyether urethane (PEU) and polycarbonate urethane (PCU); the results are expressed as the average ± standard deviation (n = 4 indents per condition for nanoindentation).

	PEU		PCU	
	No-simulant	In-simulant	No-simulant	In-simulant
Rq (nm)	4.06 ± 1.32	4.59 ± 0.72	2.83 ± 1.42	2.43 ± 1.08
Approach slope (V µm^−1^)	13.13 ± 1.41	9.79 ± 0.62	20.53 ± 3.79	16.88 ± 1.34
Retract slope (V µm^−1^)	18.69 ± 1.59	13.77 ± 0.70	32.81 ± 3.77	28.45 ± 2.29

## Discussion

In this study, we investigated potential changes in extractable and leachable profiles as well as surface characteristics of two types of polyurethane once implanted, the PEU and PCU, which are commonly used for DBS lead isolation^6^. We used a developed brain phantom conceived for leachable screening to perform the study and evaluate the impact of DBS lead implantation on the characteristics of the studied polymers. DBS leads are designed to remain implanted for the lifetime of the patient and therefore represent a chronic long-term exposure scenario^26^. However, reproducing such implantation durations experimentally is not feasible within a single study. Although the ISO 10,993–18 biocompatibility standard indicates that leachable monitoring should be performed when the quantity of any extractable material released from the medical device presents a potential safety hazard in light of its estimated clinical release, a clear solution or methodology to establish such a profile is not proposed^14^. In this context, the present work was designed as a first-step screening approach aiming to identify compounds with a potential to migrate under long-term contact conditions, rather than to directly quantify leachables in the simulant. Forced extraction experiments were deliberately employed as part of an extractables screening strategy in accordance with the recommendations of ISO 10,993–12, which require the use of both polar and non-polar solvents to maximize the identification of potentially extractable compounds from polymer-based medical devices^13^. In our case, hexane (non-polar) and acetone (polar) extractions were not intended to simulate intracerebral conditions but to establish a comprehensive worst-case extractable profile to inform subsequent leachable investigations. The extraction conditions (solvents, extraction time, and solvent-to-sample ratio) were selected based on ISO 10,993–12 recommendations and previously validated protocols for polyurethane extractables profiling an intermediate ratio (70 mg/mL) to ensure efficient compound recovery while maintaining analytical sensitivity for GC–MS, LC–MS, and ICP-OES analyses^13,27^. Importantly, all comparative analyses were performed on the same DBS lead before and after contact with the brain simulant, allowing each probe to serve as its own internal reference. This paired experimental design was intentionally selected to minimize inter-lead variability and to focus on material-specific changes induced by long-term contact rather than on inter-sample differences.

The use of a brain phantom allows controlled, reproducible and extended exposure conditions, which are difficult to achieve in biologically complex systems. Although water is the major component of the brain environment, the lipid-rich nature of brain tissue supports the relevance of using solvents less polar than water during extractables screening such as acetone, as lipophilic compounds may preferentially partition into neural tissues in vivo^28^. In our study, the 180-day duration represents a worst-case screening condition for identifying compounds with a potential to be released over extended periods, consistent with the exploratory nature of the study and the aim of prioritizing substances of interest for subsequent targeted investigations rather than establishing quantitative clinical exposure levels.

The results of this study support the decrease of polyurethane monomers in DBS probes, i.e., 4,4′-MDI and 1,4-butanediol oligomers in the case of PEU and 4,4′-MDI and 1,6-hexanediol in the case of PCU. First, we found a decrease in the 4,4′-MDI between the no-simulant and the in-simulant PEU and PCU polymers after a comparison of both extractable profiles. This decrease may reflects a change in extractable content and therefore indicates a potential release. In this regard, the use of acetone and hexane enables efficient recovery of embedded compounds from polyurethane matrices, as previously demonstrated by near-quantitative extraction yields^27^. The observed decrease was greater in the PEU than in the PCU (approximately 82% of the initial 4,4′-MDI vs 62% in the PCU). These results could explain the decrease in both the loading and unloading slope values in the nanoindentation experiments between the no-simulant and in-simulant polymers, since the 4,4′-MDI represents the hard segment of polyurethane and is responsible for the rigidity of the polymer^24^. It should be noted however that 4,4′-MDI is chemically unstable in aqueous environments and rapidly undergoes hydrolysis, leading to the formation of diamines such as 4,4′-methylenedianiline (MDA), a degradation pathway that is well documented in the literature^29^. In this study, the GC–MS analytical method employed enables the detection of MDA which would be expected to elute at a retention time of 17.770 min under the chromatographic conditions used. However, no signal corresponding to MDA was detected in the extractable profiles of the probes. This absence of detectable transformation products should be interpreted cautiously, as it may reflect concentrations below limit of detection, limited extraction efficiency under the applied conditions, preferential partitioning into the simulant or rapid chemical transformation prior to analysis. This observation suggests that, if transiently released, 4,4′-MDI and/or its degradation products may preferentially partition into the simulant rather than accumulate within the polymer or remain detectable in the extractable fraction. Although alternative explanations in mechanical changes could be related to polymer swelling or water uptake, this hypothesis is not supported by the IR spectroscopy data because of the absence of significant changes in the characteristic OH stretching vibration at approximately 3400 cm⁻^1^, which suggests that the polymer matrix did not retain detectable levels of water. Second, we detected a decrease in the number of 1,4-butandiol oligomers in PEU and 1,6-hexanediol carbonate diol oligomers in PCU between nonsimulant and in-simulant polymers. The decrease was much notable for the low n-oligomer weight, reaching 99.05% for PEU and 92.98% for PCU. These diols constitute the soft segments of the polymers, and their loss may suggest a possible release into the surrounding environment. Finally, the increase in the Al signal in the EDS analysis is probably due to the loss of oxygen and carbon signals, and the increase in roughness found in both the PEU and PCU reflects the importance of this loss^30,31^. In addition, ICP‒OES analysis favored Al loss after contact with the brain simulant in the PEU but could not be performed for the PCU. These two techniques probe complementary phenomena (surface elemental presence versus bulk extractable content) and are therefore not contradictory. Al can be added in the form of aluminum oxide to polyurethane to improve abrasion resistance, hardness, corrosion resistance, thermal stability, electrical insulation and mechanical strength^32,33^. In the IR spectroscopy analyses of both PEU and PCU, we found that no significant changes occurred between the no-simulant and in-simulant probes. The absence of significant IR changes also suggests that the observed variations in extractable profiles may not exclusively reflect bulk chemical degradation, but could also arise from surface reorganization, additive redistribution, microphase rearrangement within segmented polyurethane domains or physicochemical aging during prolonged contact with the simulant. Comparable results were reported by Christenson et al.^23^, who compared the IR spectra of the PEU and PCU after in vivo implantation for 20 weeks and 15 months and reported that no spectral changes were detected for the PCU and that a slight decrease in ether function was detected for the PEU after 20 weeks.

The low stability of PEU during aging experiments has been confirmed and well established in many studies, showing its susceptibility to temperature^34^ and lipid composition^35^. For example, Tanzi et al*.* demonstrated greater stability of PCU than PEU in a lipid dispersion made of 0.1% (w/v) cholesterol and 0.25% (w/v) phosphatidylcholine in a phosphate-buffered saline solution at pH 7.4 and in bile that contained approximately 15% (w/w) lipids (cholesterol, phospholipids, and biliary salts), 5% (dry weight) proteins, and electrolytes at pH 6.8 to 8.0^35^. Christenson et al*.* reported increased stability of PCU after subcutaneous implantation in Sprague‒Dawley rats for 15 months, but the chemical and physical degradation observed on explanted PCU surfaces indicated that the PCU was also susceptible to biodegradation^23^.

Focusing on the targeted chemicals present in DBS leads, the differences between the extractable profiles in the no-simulant and in-simulant PEU and PCU leads could imply a migration of 4,4′-MDI in the brain simulant. Although this chemical is reported to be a suspected carcinogenic component, an association between 4,4′-MDI exposure and cancer has not been established^36^. We also note that the definition of the no observed adverse effect level (NOAEL) for systemic exposure to 4,4′-MDI has not been established^37^. However, these isocyanates have been reported to have irritative and pro-inflammatory activity with increased IgE production in the skin, macrophage aggregation with reduced phagocytosis activity and yellowish inclusions, as well as concentration-dependent inflammation, which could impact the tolerability of DBS leads^38^. Furthermore, the role of the 4,4′-MDI in the development of gliosis around probes once they are implanted in the brain should be further studied. On the other hand, Aluminum was detected via ICP‒OES in acetone extract and its presence was confirmed by EDS. The chemical speciation and bioavailability of aluminum could not be determined in this study and therefore no conclusion on in vivo toxicity can be drawn. However, migrating Al was found to be more important in PEU and could result from the greater degradation of PEU after implantation than in PCU, which could increase the migration of chemicals trapped inside the insulating polymer. Although Al is generally used as aluminum oxide, which may form stable passivating or oxide-rich surface layers that limit its effective release under physiological conditions, we were not able to identify its molecular form in this study. Higher-resolution techniques such as XPS or cross-sectional TEM-EDS should be used to determine its chemical speciation and distribution within the polymer matrix^39^. In any case, Al has been reported to induce irritative effects, and its potential impact on DBS lead tolerability warrants further investigation^40^.

Within this framework, the present study should be considered as a first screening approach aimed at identifying compounds with a potential to be released under long-term contact conditions. Within the ISO 10,993 biological evaluation workflow, such screening approaches may help identify compounds requiring further toxicological assessment according to ISO 10,993–17 and may support the chemical characterization and risk management steps recommended for implantable medical devices^41^. The extrapolation of these results to clinical conditions should be performed cautiously and limited to the identification of compounds with a potential to be released rather than to biological or toxicological effects. Although all comparative analyses were performed on the same DBS lead before and after contact with the brain simulant allowing each probe to serve as its own internal reference and thereby minimizing inter-lead variability in order to focus on material-specific changes induced by prolonged contact with the simulant, it should be acknowledged that this exploratory design did not include biological or technical replicates in the conventional statistical sense, which limits the statistical robustness of quantitative comparisons. From a methodological perspective, this work relies on an indirect estimation of leachables through comparative extractable profiling rather than direct compound quantification in the simulant. From a biological perspective, the physicochemical brain simulant provides a controlled screening environment, although it does not capture cellular uptake, immune responses, active clearance or other in vivo determinants of biological fate. Accordingly, the results are interpreted in terms of qualitative trends and comparative tendencies rather than statistically validated effects. In this context, the brain simulant should be regarded as a first-line screening tool, suitable for prioritizing compounds of interest under controlled long-term contact conditions.

A comprehensive toxicological risk assessment would therefore require a dedicated analytical strategy, including targeted LC–MS/MS methods and compound-specific validation of extraction efficiency, recovery, sensitivity and stability for both 4,4′-MDI and its degradation products. Further investigations integrating biologically models such as cellular systems, dynamic environments, explanted leads or clinical samples will be required to assess biological fate, cellular uptake, immune interactions and clinical relevance of the identified leachables. In addition, the migration of compounds such as 4,4′-MDI and aluminum requires confirmation through direct analysis of the simulant following contact with the polymers. Such studies will necessitate compound-specific analytical validation including extraction efficiency, recovery and sensitivity as well as dedicated investigations focusing on release kinetics. Given the chemical instability and aqueous reactivity of 4,4′-MDI^29^, validation of both the parent compound and its degradation products will be essential. Such future investigations will require targeted analytical strategies specifically designed to monitor both release and transformation kinetics of 4,4′-MDI and MDA in the simulant. Higher-resolution analytical techniques will therefore be implemented in subsequent studies to further elucidate these aspects. Future studies should also focus on direct analysis of the simulant using targeted and validated analytical methods, determination of aluminum speciation and integration of more biologically relevant or mechanically dynamic models to better support toxicological and regulatory assessment.

## Conclusion

In this work, we identified extractables and potential leachables from PEU and PCU implantable DBS probes by comparing the extractable profiles before and after contact with a brain phantom. The extraction and quantification of 4,4′-MDI, a suspected carcinogenic chemical, revealed a remarkable decrease in both polymers after 180 days of contact but was more important in the PEU than in the PCU. We also observed signals consistent with the presence and possible redistribution of aluminum-containing additives within the polymers. While EDS revealed a higher relative aluminum signal at the surface of PCU after contact, this observation suggests a distinct surface exposure or redistribution profile of inorganic additives compared to the polymer matrix. Bulk extractable analysis by ICP-OES suggested a more pronounced aluminum loss in PEU, which may be consistent with its higher susceptibility to degradation compared with PCU. These results need to be strengthened by analyzing leachables directly in the brain phantom to confirm those results. Future studies combining direct simulant analysis with targeted methods addressing transformation pathways and release kinetics are therefore required before any toxicological extrapolation can be made.

## Materials and methods

### Samples and study design

Two DBS leads of two different types of polyurethane were used in this study: polyetherurethane 80A (reference 3389; Medtronic; Minneapolis, MN, USA) and polycarbonate urethane 55D Bionate® (Infinity DBS reference 6171; Abbott Neuromodulation; Plano, TX, USA).

**Fig. 5:**
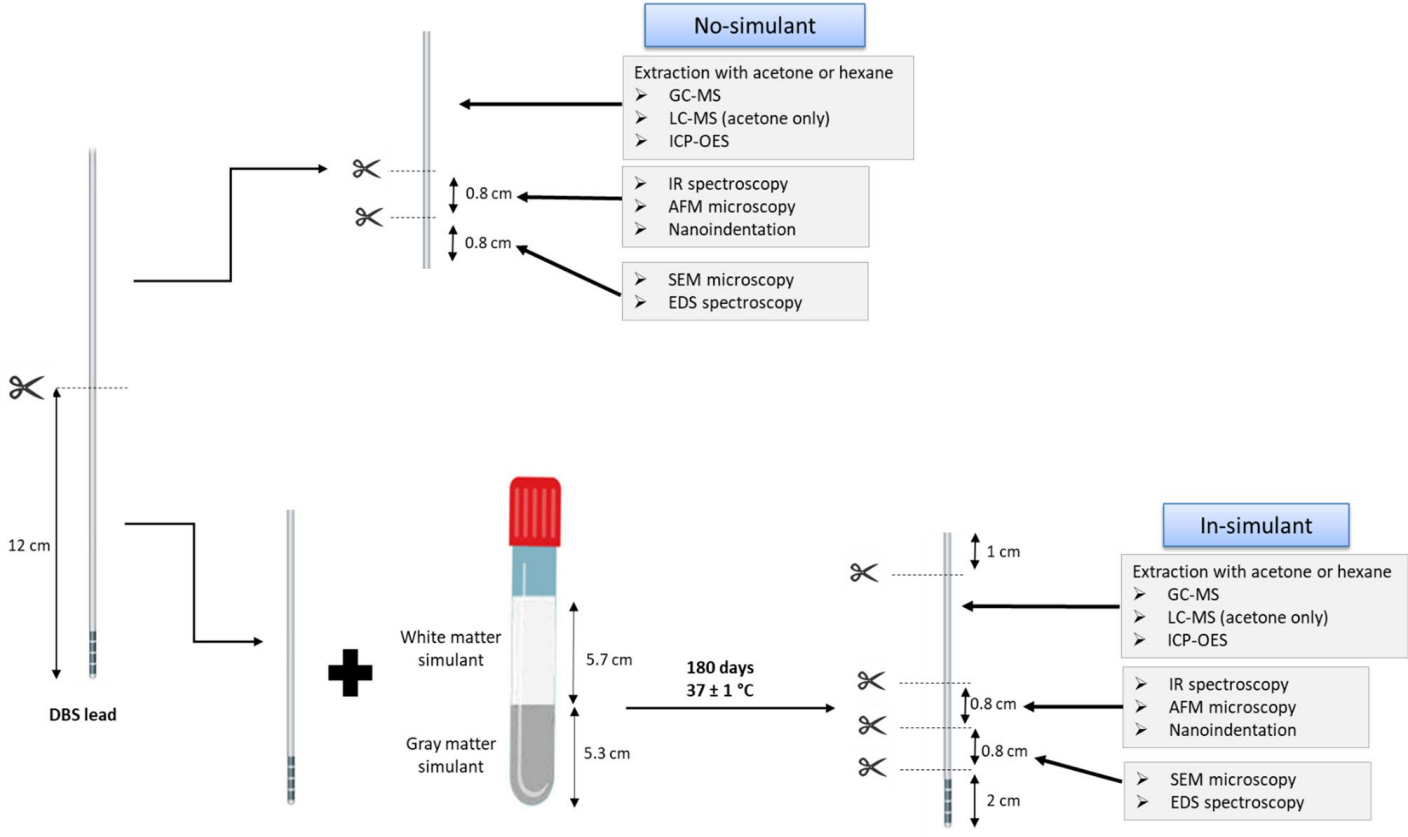
Study design of leads without contact with the simulant (no-simulant) and after 180 days of incubation with the simulant (in-simulant): GC‒MS, gas chromatography–mass spectrometry; LC‒MS, liquid chromatography–mass spectrometry; ICP‒OES, inductively coupled plasma optical emission spectroscopy; IR, infrared; AFM, atomic force microscopy; SEM, scanning electron microscopy; EDS, energy-dispersive X-ray spectroscopy.

The 12-cm distal part of each lead was studied without placement in a brain simulant (no-simulant) and after 180 days in a simulant (in-simulant). The simulant was a brain phantom whose composition was already detailed^42^. The stay of 180 days at 37 °C was realized in a glass tube of 23 mL capacity (Ref TV5002, ThermoFisher Scientific, Courtaboeuf Cedex, France) containing a brain phantom for leachable screening with white matter and gray matter simulants (Fig. 5). To ensure consistent analytical conditions, samples from no-simulant and in-simulant samples were analyzed on the same day to minimize the risk of intraday variability.

Sample profiling after extraction with hexane (Chromasolv™, Honeywell, Roissy CDG, France) and acetone (Pestipur®, Carlo Erba, Val-de-Reuil, France) was performed on both parts of the same leads for comparison to avoid intrabatch variabilities, as described previously Fig. 5. Once identified, all the components were compared to the list of carcinogenic, mutagenic, or toxic reproductive substances (CMR substances) of the European Regulation No. 1272/2008, known as CLPs for the classification, labeling and packaging of substances and mixtures^43^, and to the chemical neurotoxic agents provided in the Encyclopedia of Occupational Health and Safety of the International Labor Organization^44^. Estimation of leaching of CMR or neurotoxic components is based on the difference between extractable profiles in both parts (before and after contact with the brain phantom), which are quantified once their analytical signal is found to be more than 10 times the signal noise (S/N) of the baseline. To explain any eventual differences in leachate migration between both the PEU and PCU, surface characterization was performed before and after contact with the brain phantom.

### Extractable profiling and leachable estimation

#### Extraction procedure

Extractions were made following the recommendations of ISO 10,993 parts 12 and 18 of the biocompatibility standards^13^. All devices were weighed and extracted in two different solvents of different polarities, n-hexane (nonpolar) and acetone (polar), which are considered fatty acid representatives^45^. Each device was extracted with a ratio of 70 mg per mL of pure solvent. Extraction was accomplished by adding a single device to an appropriate extraction type I glass vessel with the corresponding solvent, which was then placed in an oven at 37 °C and shaken every 24 h for 72 h. At the end of the extraction, each extract solution was analyzed in triplicate (three repeated analytical measurements of the same extract solution) by ICP–OES for inorganic extractables, GC–MS for volatile and semivolatile extractables, and LC–MS for nonvolatile extractables. Solvent blank injections were performed between samples and no residual peaks attributable to carry-over were observed. To ensure analytical consistency, all analyses were performed within the same analytical sequence on the using identical instrumental parameters, with solvent blanks and quality control injections included throughout the run to monitor analytical stability.

#### Gas chromatography–mass spectrometry

Direct injections of 1 µL of hexane and acetone extraction solvents were performed via an Agilent 5977/B GC‒MS chromatograph (Agilent, Les Ulis, France) equipped with electronic impact ionization tuned to 70 eV and an Agilent HP‒5MS 19091S‒433UI (5%-phenyl)-methylpolysiloxane phase capillary column (30 m × 0.25 mm × 0.25 µm) capillary column (Agilent, Les Ulis, France). The oven temperature curve started at 100 °C for 4 min and then rose to 160 °C at a rate of 15 °C/min, was maintained at this temperature for 4 min, then rose again to 300 °C at a rate of 20 °C/min and was maintained at this temperature for 21 min. The total analysis time was 40 min. The N55 helium speed in the column was 0.6 mL/min. The split ratio was set to 25:1; the injector temperature was 250 °C, the MS temperature was 230 °C, the transfer line temperature was 300 °C, and the quadrupole temperature was 150 °C.

Extractable identification was performed in scan mode with screening of the m/z fragments from 50 to 550 with a scan speed of 2.9 scans per second, and the results were compared to analytical standards: 4,4′-MDI (Ref 33,428-100MG-R, Sigma-Aldrich; MC2, Clermont-Ferrand, France); 4,4′-MDA (ref 31,640-250MG, Sigma-Aldrich; MC2, Clermont-Ferrand, France); and 14 certified reference components present in *Trace*CERT® (ref 01,829-1 mL; Sigma-Aldrich Production GmbH, Switzerland), and also compared with those of the National Institute of Standards and Technology (NIST2020) mass spectra library and MassHunter Unknown analysis software (version 10.2, Agilent, Les Ulis, France). Identification levels (confirmed, confident, tentative and unknown) were defined according to USP < 1663 > recommendations.

To quantify 4,4′-MDI, a stock solution of 4,4′-MDI (Sigma‒Aldrich, France) was prepared at 10 mg/ml in acetone. Standards and quality control samples were prepared by diluting the appropriate quantity of stock solution in acetone. Five-point calibration curves were constructed in the concentration range of 0.1–2.5 mg/ml in which a quadratic fit was weighted via the extracted ion chromatogram at m/z 231. M/z 180 and 208 were used as qualifier ions. The limits of quantification (LOQs) and detection (LODs) were estimated to be 4.5 µg/ml and 1.4 µg/ml, respectively. Quantification was performed via MassHunter Quantitative analysis software (version 10.2; Agilent, Les Ulis, France). The results are expressed as percentages (w/w) of the extracted medical device.

#### Liquid chromatography‒mass spectrometry

The analyses were carried out on an Ultimate® 3000 RSLC UHPLC chain (ThermoScientific) equipped with a Kinetex® EVO C18 column (100 × 2.1 mm; 1.7 µm) (Phenomenex, Le Pecq, France) in an oven set at 30 °C. The mobile phase consisted of water containing 0.1% (v/v) formic acid (Phase A) and acetonitrile containing 0.1% (v/v) formic acid (Phase B), the gradient variation of which is described in Table 1. The flow rate of the mobile phase was set at 0.45 ml/min. The injection volume was 5 µl.

Detection was performed via an Orbitrap Q Exactive mass spectrometry detector (ThermoScientific) with the following parameters: ionization using electrospray positive and negative modes, m/z ratio ranging from 80 to 1200, resolution at 35,000, spray voltage at 3.2 (positive) and 3.0 (negative), maximum IT: 50 ms, AGC target: 1e6, capillary temperature: 320 °C, and auxiliary gas temperature: 400 °C. The “sheath gas”, auxiliary gas and “sweep gas” flow rates were 50, 10, and 2 (units/N2), respectively. Analyses were only performed on acetone extraction solution since hexane was not adapted to the mobile phase composition. LC‒MS chromatograms were analyzed via XCalibur Qual Browser analysis software (version 4.0.27.19; ThermoScientific). Since no specific database is available for this type of analysis, the m/z values of all the peaks were compared with those of the compounds reported by Hooper et al. when other DBS leads were analyzed^23^.

#### Inductively coupled plasma optical emission spectroscopy ICP‒OES

We used ICP‒OES (iCAP PRO XP Duo® THERMO FISHER) for the screening and quantification of 34 elements: Ag, Al, As, B, Ba, Be, Bi, Ca, Cd, Co, Cr, Cu, Fe, Ir, K, Li, Mg, Mn, Mo, Na, Ni, Pb, Pt, Rh, Ru, Sb, Se, Sn, Sr, Te, Ti, Tl, V, Zn. Since acetone and hexane are not suitable for ICP‒OES detection because of their high organic composition, the quantification of each element was validated after the evaporation of solvents under the laminar air flow of an ISO 4.8 microbiological safety cabinet, and the recovery process of the residual elements was performed with 6 mL of 1% nitric acid solution (Sigma‒Aldrich, France). Recovery validation was assessed through recovery experiments performed by an ISO 17,025 accredited external laboratory (DORAN LAB, Toussieu, France). Recovery of aluminum and copper was evaluated at three concentration levels (low: 10 µg mL^−1^, medium 100 µg mL^−1^ and high 1000 µg mL^−1^) by spiking blank matrices of acetone and hexane. Mean recoveries ranged from 95 to 111%, demonstrating the accuracy of the ICP-OES method (Supplementary material 5). All measurements were performed within a single analytical run on the same day, and instrumental drift was monitored through calibration verification, internal standard (yttrium) monitoring, and quality control samples in accordance with ISO 11,885:2009 standard. Each element was identified and quantified via two specific line spectra, and the results are expressed as the weight of the elements per weight of the analyzed medical device.

### Spectral and surface analysis

The characterization of the PEU and PCU surfaces without and after contact with the brain phantom was performed on 10 points of each analyzed part without prior treatment. To detect changes in the polymer structure, such as hydrolysis, oxidation or possible water impregnation, infrared (IR) spectroscopy was performed via a Nicolet 5700® (Thermo Electron) spectrometer in attenuated total reflectance mode. A total of 128 scans with a 4 cm^−1^ resolution were collected to acquire each spectrum between 400 and 4000 cm^−1^.

Changes in the surface visual aspects (such as cracks or roughness) and composition at the surfaces of the PEU and PCU samples were also investigated (n ≥ 6 surface locations per condition) by SEM using a field emission scanning electron microscope (Regulus 8230, Hitachi, Japan). SEM observations were performed at an accelerating voltage of 1 kV using a secondary electron detector. For EDS microanalysis, an accelerating voltage of 10 kV was applied, using an Ultim Max 170 mm^2^ detector coupled with AZtec software (Oxford Instruments, UK). The samples were directly deposited on aluminum stubs using conductive adhesive carbon tabs. No additional surface cleaning, washing, drying or conductive coating (e.g. gold or carbon sputtering) was applied before analysis. This approach was deliberately chosen to preserve the native surface state of the polymers after contact with the brain simulant and to avoid any modification or removal of loosely bound surface species. The analyses were performed under low-vacuum conditions compatible with non-conductive polymeric materials.

To quantify the roughness changes in the polymer surfaces, we used atomic force microscopy (AFM) measurements with an Innova® AFM (Bruker, Palaiseau, France). Images and roughness data were gathered in tapping mode in air with a silicium probe (RTESPA-300 from Bruker; resonant frequency: 300 kHz; elasticity modulus range: 0.2–2 GPa): phase and topographic images were both exploited. In addition, nanoindentation assays (n = 4 indents per condition for nanoindentation) were performed to detect any changes in the approach and retract the slope of the polymer, implying any change in composition.

## Supplementary Information

Below is the link to the electronic supplementary material.

Supplementary Material 1.

## Data Availability

The datasets used and/or analysed during the current study available from the corresponding author on reasonable request.
