# Supplementary material for: Screening for compounds released from deep brain stimulation probes by contact with a brain simulant
Source: Sci Rep. 2026 Apr 9;16:16677. doi: 10.1038/s41598-026-46292-5 (PMC13219594; doi:10.1038/s41598-026-46292-5)
Supplement: Supplementary file 1 — Supplementary Material 1. [file 41598_2026_46292_MOESM1_ESM.docx]

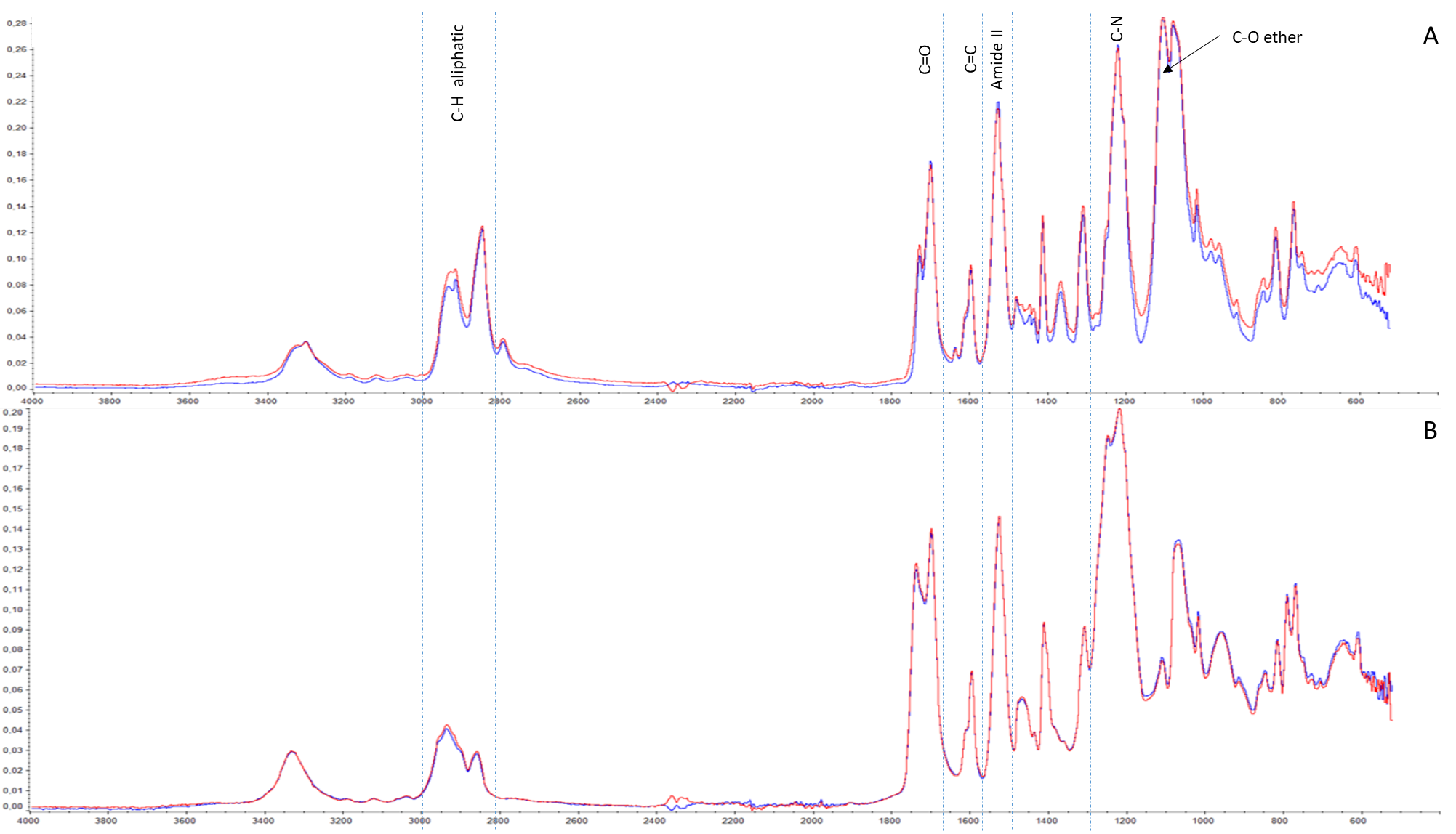


Supplementary material 1 Fourier transform infrared power spectra of the nonsimulant (red) and in-simulant (blue) polyether urethane (A) and polycarbonate urethane (B)


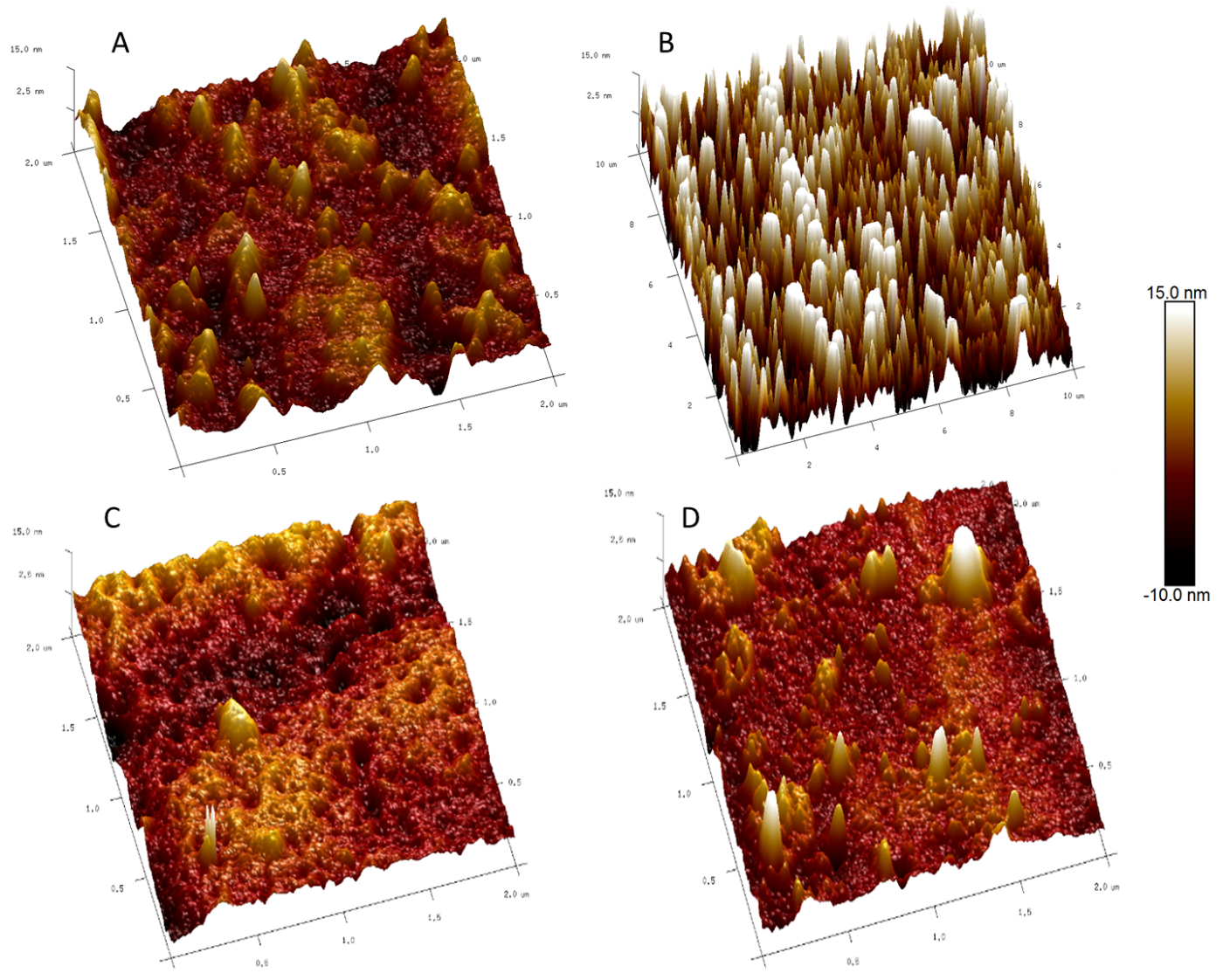


Supplementary material 2 Atomic force microscopy images of the evolution of the polymer surface: between (A) no-simulant and (B) in-simulant polyether urethane and between (C) no-simulant and (D) in-simulant polyether urethane.

*Supplementary material 3 List of extractables identified in polyether urethane and polycarbonate urethane DBS probes via gas chromatography–mass spectrometry via the National Institute of Standards and Technology (NIST 2020) database; CMR: Carcinogenic, mutagenic or reprotoxic.*

| **Chemicals in acetone and hexane extraction of Polyether urethane** | | | | | |
| --- | --- | --- | --- | --- | --- |
| **Compound RT** | **Compound Name** | **Identification level** | **CAS Number** | **Extraction solvant** | **CMR or neurotoxic (Y/N)** |
| 16.6 | Benzene, 1,1'-[1,2-ethanediylbis(oxy)]bis- | tentative | 104-66-5 | Hexane | N |
| 17.07 | Polyether polyols-derivatives | tentative |  | Acetone | N |
| 18.09 | Methylene diphenyl diisocyanate other isomers | confident |  | Acetone | Y |
| 18.23 | Methylene diphenyl diisocyanate other isomers | confident |  | Acetone | Y |
| 18.34 | Polyether polyols-derivatives | tentative |  | Acetone, hexane | N |
| 18.52 | 4,4'-Methylene diphenyl diisocyanate | confirmed | 101-68-8 | Acetone, hexane | Y |
| 18.59 | Methylene diphenyl diisocyanate other isomers | confident |  | Acetone, hexane | Y |
| 19.68 | Polyether polyols-derivatives | tentative |  | Acetone | N |
| 20.01 | Polyether polyols-derivatives | tentative |  | Hexane | N |
| 20.80 | Polyether polyols-derivatives | tentative |  | Acetone, hexane | N |
| 22.77 | Polyether polyols-derivatives | tentative |  | Acetone | N |
| 24.68 | Polyether polyols-derivatives | tentative |  | Acetone, hexane | N |
| 29.48 | Polyether polyols-derivatives | tentative |  | Acetone | N |
| 33.08 | Polyether polyols-derivatives | tentative |  | Acetone, hexane | N |
| 37.29 | Irganox 1076 | confirmed | 2082-79-3 | Acetone, hexane | N |

| **Chemicals in acetone and hexane extraction of Polycarbonate urethane** | | | | | |
| --- | --- | --- | --- | --- | --- |
| **Compound RT** | **Compound Name** | **Identification level** | **CAS Number** | **Extraction solvant** | **CMR or neurotoxic (Y/N)** |
| 7.48 | 1-Propanone, 1-(3-pyridinyl)- | tentative | 1570-48-5 | Acetone | N |
| 13.27 | 2,6-Bis(1,1-dimethylethyl)-4-methyl-4-isopropylcyclohexa-2,5-dien-1-one | tentative | 4278-82-4 | Acetone, hexane | N |
| 14.89 | Polycarbonate polyols-derivatives | tentative |  | Acetone | N |
| 15.67 | 4-tert-Octylphenol | tentative | 140-66-9 | Acetone | N |
| 15.73 | 1-Hepten-4-ol | tentative | 3521-91-3 | Acetone | N |
| 15.75 | 4-(7-Methyloctyl)phenol | tentative | 24518-48-7 | Hexane | N |
| 16.08 | Benzene, 1,1'-(1-methylethylidene)bis[4-methyl- | confident | 1000400-89-0 | Acetone, hexane | N |
| 16.41 | Benzene, 1,1'-(3-methyl-1-propene-1,3-diyl)bis- | confident | 7614-93-9 | Hexane | N |
| 16.71 | Polycarbonate polyols-derivatives | tentative |  | Acetone | N |
| 17.31 | Polycarbonate polyols-derivatives | tentative |  | Acetone | N |
| 18.09 | Methylene diphenyl diisocyanate other isomers | confident |  | Acetone | Y |
| 18.52 | 4,4'-Methylene diphenyl diisocyanate | confirmed | 101-68-8 | Acetone, hexane | Y |
| 18.59 | Methylene diphenyl diisocyanate other isomers | confident |  | Acetone | N |
| 18.59 | Polycarbonate polyols-derivatives | tentative |  | Acetone | N |
| 20.01 | Polycarbonate polyols-derivatives | tentative |  | Acetone | N |
| 20.56 | Bis(2-ethylhexyl) phthalate | confirmed | 117-81-7 | Hexane | Y |
| 20.95 | Ethanol, 2-(1-methylethoxy)- | tentative | 109-59-1 | Acetone | N |
| 23.58 | Polycarbonate polyols-derivatives | tentative |  | Acetone | N |
| 24.99 | Polycarbonate polyols-derivatives | tentative |  | Acetone | N |
| 25.93 | Polycarbonate polyols-derivatives | tentative |  | Acetone | N |
| 28.29 | Polycarbonate polyols-derivatives | tentative |  | Acetone | N |
| 30.69 | Polycarbonate polyols-derivatives | tentative |  | Acetone | N |


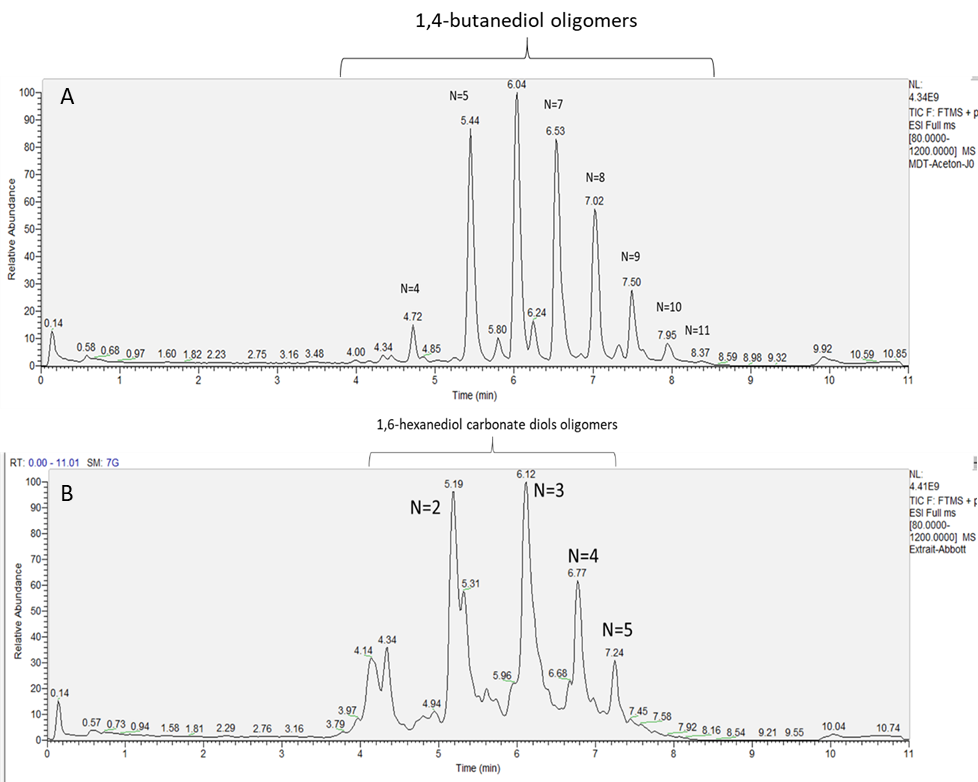


Supplementary material 4 Chromatograms of acetone extracts of no-simulant (A) polyether urethane and (B) polycarbonate urethane leads, N: number of monomers.

*Supplementary material 5*

*Validation of the evaporation and recovery procedure was performed to assess potential elemental losses, matrix effects and repeatability for aluminum (Al) and copper (Cu) as Hexane and acetone samples doped with certified multi-element standards (ref 12956213; Fisher Chemical™ ; France) to obtain three concentrations of 10; 100 and 1000 µg/L. Solvents were evaporated to dryness under hood in polypropylene containers, followed by reconstitution in 1% nitric acid and ICP-OES analysis in triplicate. The calculated recovery from each concentration for each element is presented in the table below*

| *Element* | *solvent* | *concentration* | *Recovery* |
| --- | --- | --- | --- |
| *Al* | *Acetone* | *10* | *100 %* |
| *Al* | *Acetone* | *100* | *97 %* |
| *Al* | *Acetone* | *1000* | *98 %* |
| *Al* | *Hexane* | *10* | *111 %* |
| *Al* | *Hexane* | *100* | *105 %* |
| *Al* | *Hexane* | *1000* | *97 %* |
| *Cu* | *Acetone* | *10* | *103 %* |
| *Cu* | *Acetone* | *100* | *102 %* |
| *Cu* | *Acetone* | *1000* | *99 %* |
| *Cu* | *Hexane* | *10* | *95 %* |
| *Cu* | *Hexane* | *100* | *101 %* |
| *Cu* | *Hexane* | *1000* | *97 %* |
